# Cardiovascular disease mortality based on verbal autopsy in low- and middle-income countries: a systematic review

**DOI:** 10.2471/BLT.23.289802

**Published:** 2023-07-05

**Authors:** Ajay Acharya, Hafizur Rahman Chowdhury, Zulfikar Ihyauddin, Pasyodun Koralage Buddhika Mahesh, Tim Adair

**Affiliations:** a The Nossal Institute for Global Health, Melbourne School of Population and Global Health, University of Melbourne, Level 5, 333 Exhibition St, Melbourne, Victoria, 3000 VIC, Australia.; b CDC Foundation, Melbourne, Australia.

## Abstract

**Objective:**

To conduct a systematic review of verbal autopsy studies in low- and middle-income countries to estimate the fraction of deaths due to cardiovascular disease.

**Method:**

We searched MEDLINE®, Embase® and Scopus databases for verbal autopsy studies in low- and middle-income countries that reported deaths from cardiovascular disease. Two reviewers screened the studies, extracted data and assessed study quality. We calculated cause-specific mortality fractions for cardiovascular disease for each study, both overall and according to age, sex, geographical location and type of cardiovascular disease.

**Findings:**

We identified 42 studies for inclusion in the review. Overall, the cardiovascular disease cause-specific mortality fractions for people aged 15 years and above was 22.9%. This fraction was generally higher for males (24.7%) than females (20.9%), but the pattern varied across World Health Organization regions. The highest cardiovascular disease mortality fraction was reported in the Western Pacific Region (26.3%), followed by the South-East Asia Region (24.1%) and the African Region (12.7%). The cardiovascular disease mortality fraction was higher in urban than rural populations in all regions, except the South-East Asia Region. The mortality fraction for ischaemic heart disease (12.3%) was higher than that for stroke (8.7%). Overall, 69.4% of cardiovascular disease deaths were reported in people aged 65 years and above.

**Conclusion:**

The burden of cardiovascular disease deaths outside health-care settings in low- and middle-income countries is substantial. Increasing coverage of verbal autopsies in these countries could help fill gaps in cardiovascular disease mortality data and improve monitoring of national, regional and global health goals.

## Résumé

**Objectif:**

Mener une revue systématique des études d'autopsie verbale dans les pays à revenu faible et intermédiaire afin d'estimer la part des décès causés par des maladies cardiovasculaires.

**Méthodes:**

Nous avons exploré les bases de données MEDLINE®, Embase® et Scopus à la recherche d'études d'autopsie verbale signalant des décès liés à une maladie cardiovasculaire dans les pays à revenu faible et intermédiaire. Deux réviseurs ont passé ces études au crible, en ont extrait des informations et ont évalué leur qualité. Pour chaque étude, nous avons calculé la part de mortalité par cause pour les maladies cardiovasculaires, tant de manière globale qu'en fonction de l'âge, du sexe, de la situation géographique et du type de maladie cardiovasculaire.

**Résultats:**

Nous avons identifié 42 études à inclure dans la revue. Dans l'ensemble, la part de mortalité par cause pour les maladies cardiovasculaires s'élevait à 22,9% pour les personnes âgées de 15 ans et plus. Cette part était généralement plus importante chez les hommes (24,7%) que chez les femmes (20,9%), mais le schéma variait selon les régions de l'Organisation mondiale de la Santé. C'est dans la Région du Pacifique occidental que le plus haut taux de mortalité cardiovasculaire a été observé (26,3%), puis dans la Région d'Asie du Sud-Est (24,1%) et dans la Région africaine (12,7%). La part de mortalité due aux maladies cardiovasculaires était plus grande au sein des populations urbaines plutôt que rurales dans toutes les régions, à l'exception de la Région d'Asie du Sud-Est. La part de mortalité liée aux cardiopathies ischémiques (12,3%) était supérieure à celle des AVC (8,7%). Au total, 69,4% des décès cardiovasculaires ont été constatés chez des personnes âgées de 65 ans ou plus.

**Conclusion:**

L'impact des décès causés par une maladie cardiovasculaire en dehors des structures de santé dans les pays à revenu faible et intermédiaire est considérable. Étendre la couverture des autopsies verbales dans ces pays pourrait contribuer à combler les lacunes dans les données sur la mortalité cardiovasculaire et à améliorer le suivi des objectifs nationaux, régionaux et mondiaux en matière de santé.

## Resumen

**Objetivo:**

Llevar a cabo una revisión sistemática de los estudios sobre autopsias verbales de países con ingresos medios y bajos para estimar la proporción de fallecimientos causados por enfermedades cardiovasculares.

**Métodos:**

Buscamos en bases de datos como MEDLINE®, Embase® y Scopus para acceder a estudios sobre autopsias verbales de países con ingresos medios y bajos que hubieran registrado fallecimientos causados por enfermedades cardiovasculares. Dos revisores analizaron los estudios, extrajeron datos y evaluaron la calidad de dichos estudios. Calculamos las fracciones de mortalidad por enfermedad cardiovascular como causa específica en cada uno de los estudios, de manera general y según la edad, el sexo, la ubicación geográfica y el tipo de enfermedad cardiovascular.

**Resultados:**

Seleccionamos 42 estudios para incluirlos en la revisión. En general, las fracciones de mortalidad por enfermedad cardiovascular como causa específica en personas con una edad de 15 años o superior fueron del 22,9%. Por lo general, esta fracción fue más elevada en el caso de los hombres (24,7%) que en el de las mujeres (20,9%), pero el patrón varió entre las diferentes regiones de la Organización Mundial de la Salud. La fracción más alta de mortalidad por enfermedad cardiovascular se registró en la Región del Pacífico Occidental (26,3%), seguida de la Región de Asia Sudoriental (24,1%) y la Región de África (12,7%). La fracción de mortalidad por enfermedad cardiovascular fue más elevada en las poblaciones urbanas que en las rurales en todas las regiones, excepto en la Región de Asia Sudoriental. La fracción de mortalidad por cardiopatía isquémica (12,3%) fue superior a la de ictus (8,7%). En general, el 69,4% de los fallecimientos por enfermedad cardiovascular se registró en personas de 65 años o más.

**Conclusión:**

La carga de fallecimientos por enfermedad cardiovascular fuera de los centros de atención sanitaria en los países con ingresos medios y bajos es considerable. El aumento de la cobertura de las autopsias verbales en estos países podría ayudar a subsanar la falta de datos sobre mortalidad por enfermedad cardiovascular, y mejorar el control de los objetivos de salud a nivel nacional, regional y mundial.

## ملخص

الغرض إجراء مراجعة منهجية لدراسات التشريح السردي في الدول ذات الدخل المنخفض والدخل المتوسطة، لتقدير نسبة الوفيات الناتجة عن أمراض القلب والأوعية الدموية.

الطريقة قمنا بالبحث في قواعد بيانات MEDLINE®‎، وEmbase®‎، وScopus، عن دراسات التشريح السردي في الدول ذات الدخل المنخفض والدخل المتوسطة الدخل، والتي أبلغت عن الوفيات ناتجة عن أمراض القلب والأوعية الدموية. قام اثنان من المراجعين بفحص الدراسات، واستخراج البيانات، وتقييم جودة الدراسة. قمنا بحساب نسب الوفيات الناتجة بشكل محدد عن أمراض القلب والأوعية الدموية لكل دراسة، سواء بوجه عام ووفقًا للعمر، والجنس، والموقع الجغرافي، ونوع مرض القلب والأوعية الدموية.

النتائج قمنا بتحديد 42 دراسة لإدراجها في المراجعة. بوجه عام، كانت نسب الوفيات الناتجة بشكل محدد عن أمراض القلب والأوعية الدموية للأشخاص الذين تبلغ أعمارهم 15 عامًا وأكبر، هي %22.9. كانت هذه النسبة أعلى بشكل عام للذكور (%24.7) عن الإناث (%20.9)، لكن النمط اختلف عبر مناطق منظمة الصحة العالمية. تم الإبلاغ عن أعلى نسبة وفيات بأمراض القلب والأوعية الدموية في منطقة غرب المحيط الهادئ (%26.3)، وتليها منطقة جنوب شرق آسيا (%24.1)، والإقليم الأفريقي (%12.7). كانت نسبة الوفيات الناتجة عن أمراض القلب والأوعية الدموية أعلى في التجمعات السكانية الحضرية عنها في التجمعات السكانية الريفية في جميع المناطق، باستثناء إقليم جنوب شرق آسيا. كانت نسبة الوفيات بسبب مرض القلب الإقفاري (%12.3) أعلى من نسبة الوفيات بسبب السكتة الدماغية (%8.7). بشكل عام، تم الإبلاغ عن %69.4 من وفيات أمراض القلب والأوعية الدموية لدى أشخاص تبلغ أعمارهم 65 عامًا فما فوق.

الاستنتاج إن عبء الوفيات الناتجة عن أمراض القلب والأوعية الدموية خارج أماكن الرعاية الصحية، في الدول ذات الدخل المنخفض والدخل المتوسط، هو عبء ضخم. إن زيادة تغطية التشريح السردي في هذه الدول يمكن أن تساعد على سد الفجوات في بيانات الوفيات الناتجة عن أمراض القلب والأوعية الدموية، وتحسين رصد الأهداف الصحية الوطنية والإقليمية والعالمية.

## 摘要

**目的:**

旨在对中低收入国家的死因推断研究进行系统评价，以估算心血管疾病死亡率。

**方法:**

我们搜索了 MEDLINE®、Embase® 和 Scopus 数据库，以查询报告心血管疾病死亡情况的中低收入国家死因推断研究。两名评审员对这类研究进行了筛选、提取了数据并评估了研究质量。我们基于每项研究计算了心血管疾病病因特异性死亡率，包括总体死亡率以及按年龄、性别、地理位置和心血管疾病类型分别计算的死亡率。

**结果:**

我们确定了 42 项研究并将其纳入系统评价。总体而言，15 岁及以上人群的心血管疾病病因特异性死亡率为 22.9%。男性 (24.7%) 的这一比例通常高于女性 (20.9%)，但世界卫生组织各地区的情况各不相同。西太平洋地区 (26.3%) 报告的心血管疾病死亡率最高，其次是东南亚地区 (24.1%) 和非洲地区 (12.7%)。除东南亚地区以外，其他所有地区城市人口的心血管疾病死亡率均高于农村人口。缺血性心脏病死亡率 (12.3%) 高于中风死亡率 (8.7%)。总体而言，根据报告，69.4% 的心血管疾病死亡病例为 65 岁及以上人群。

**结论:**

在中低收入国家，医疗机构范围以外的心血管疾病死亡负担非常巨大。扩大这些国家的死因推断的覆盖范围可能有助于填补心血管疾病死亡率数据方面的空缺，并加强对国家、区域和全球健康目标的监测。

## Резюме

**Цель:**

Провести систематический обзор исследований вербальных аутопсий в странах с низким и средним уровнем дохода для оценки доли смертей от сердечно-сосудистых заболеваний.

**Методы:**

Был проведен поиск в базах данных MEDLINE®, Embase® и Scopus по исследованиям вербальных аутопсий в странах с низким и средним уровнем дохода, в которых сообщалось о случаях смерти от сердечно-сосудистых заболеваний. Два рецензента отбирали исследования, извлекали данные и оценивали качество исследований. По результатам каждого исследования была рассчитана доля смертности от сердечно-сосудистых заболеваний, обусловленная конкретной причиной, как в целом, так и в зависимости от возраста, пола, географического положения и типа сердечно-сосудистого заболевания.

**Результаты:**

Было определено 42 исследования для включения в обзор. В целом доля смертности от сердечно-сосудистых заболеваний, обусловленная конкретной причиной, среди людей в возрасте 15 лет и старше составила 22,9%. Как правило, эта доля была выше у мужчин (24,7%), чем у женщин (20,9%). Однако в разных регионах Всемирной организации здравоохранения она была разной. Самая высокая доля смертности от сердечно-сосудистых заболеваний была зарегистрирована в регионе Западной части Тихого океана (26,3%), за ним следуют регион Юго-Восточной Азии (24,1%) и регион Африки (12,7%). Доля смертности от сердечно-сосудистых заболеваний была выше среди городского населения по сравнению с сельским во всех регионах, за исключением региона Юго-Восточной Азии. Доля смертности от ишемической болезни сердца (12,3%) превышала долю смертности от инсульта (8,7%). В целом 69,4% смертей от сердечно-сосудистых заболеваний приходилось на людей в возрасте 65 лет и старше.

**Вывод:**

В странах с низким и средним уровнем дохода число смертей от сердечно-сосудистых заболеваний вне медицинских учреждений является значительным. Расширение охвата вербальными аутопсиями в этих странах может помочь заполнить пробелы в данных о смертности от сердечно-сосудистых заболеваний и способствовать улучшению мониторинга национальных, региональных и глобальных целей в области здравоохранения.

## Introduction

Cardiovascular disease is the largest cause of death due to noncommunicable disease globally. Data from the Global Burden of Disease (GBD) indicate that cardiovascular disease caused 18.5 million deaths worldwide in 2019, which corresponded to about 44% of all noncommunicable disease deaths.^1^ These deaths occurred predominantly in people aged 70 years and older and were mainly due to ischaemic heart disease or stroke, for which the main preventable risk factors are high blood pressure, high blood sugar and cholesterol levels, obesity, air pollution, tobacco and poor diet.^1^^–^^3^ Reportedly, 57% of premature deaths due to cardiovascular diseases in 2019 occurred in low- and middle-income countries, many of which are progressing through the epidemiological transition, and are experiencing a decline in infectious disease mortality along with a concurrent growth in cardiovascular disease mortality.^1^^,^^2^ Hence, one target of the sustainable development goals is to reduce premature cardiovascular disease deaths by one third of the level recorded in 2015.^4^

In many low- and middle-income countries, however, the burden of cardiovascular disease mortality is unclear because civil registration and vital statistics systems are poor, and because accurate data on the cause of death is mostly unavailable outside health-care settings.^5^^–^^7^ As a result, estimates of the cause of death in these countries have relied heavily on the modelling of data from the World Health Organization (WHO) and GBD studies. Furthermore, as the data available on cardiovascular disease mortality are limited, these estimates have wide uncertainty intervals. Moreover, the actual prevalence may have been underestimated and, consequently, understanding of the burden of cardiovascular disease in many populations may be inadequate.^2^

Verbal autopsy is the recommended method for providing routine information on the cause of death in low- and middle-income countries with low-quality or non-existent civil registration and vital statistics systems, and low coverage of medical certification of the cause of death.^8^ The prime objective of verbal autopsy is to provide population estimates of the fraction of deaths due to different causes in places where a high proportion of people die at home.^9^ Health and Demographic Surveillance System sites and epidemiological research have used verbal autopsy methods for over 50 years and these methods are increasingly being used as part of routine surveillance by civil registration and vital statistics systems.^9^^,^^10^ In a verbal autopsy, an interviewer collects information on signs and symptoms and on any health care sought during the illness that led to a person’s death, by questioning a close relative of the deceased person using a standardized questionnaire.^9^ The most likely cause of death is assigned on the basis of the information collected either by physician-certified verbal autopsy, where at least two physicians review the information and disagreement is resolved by consensus or by a third physician, or by computer-coded verbal autopsy, which uses data-driven algorithms or diagnostic criteria developed by experts.^11^ The use of verbal autopsy varies within regions and across countries. In 2022, a report by WHO’s verbal autopsy reference group revealed that the method had been implemented in several low- and middle-income countries, the majority of which were in sub-Saharan Africa and South Asia.^9^ As many countries in these regions do not have adequate death registration systems, verbal autopsies often provide the only source of information on mortality and the cause of death.^9^^,^^10^ In contrast, countries and regions with good civil registration and vital statistics systems, such as the Americas, Australasia and Europe, rely less on verbal autopsy.^5^

Systematic reviews of mortality due to specific causes based on verbal autopsy studies are sparse. The aims of our systematic review of verbal autopsy studies were to estimate the fraction of deaths in low- and middle-income countries caused by cardiovascular disease and to describe how this fraction varies by age, sex, geographical location and type of cardiovascular disease. 

## Methods

All cross-sectional and surveillance studies (e.g. prospective monitoring studies from Health and Demographic Surveillance System sites) that reported deaths from cardiovascular diseases as ascertained by verbal autopsy in low- and middle-income countries were eligible for inclusion in the systematic review. We excluded: (i) studies conducted in specific groups (e.g. infants, females or stroke survivors); (ii) studies on validity, reliability or feasibility; (iii) pilot studies; (iv) maternal mortality and stillbirth studies; and (v) studies in which the study period overlapped with another study in the same country. Full details of all inclusion and exclusion criteria are available from the data repository.^12^ We used Preferred Reporting Items for Systematic Reviews and Meta-Analyses checklists for this systematic review, and we developed the protocol and published it in the International Prospective Register of Systematic Reviews.^13^^,^^14^

### Search strategy

The search strategy was devised with the support of a University of Melbourne librarian. We converted the research question into the PICO (i.e. population, intervention, comparator and outcome) format to identify keywords.^15^ Then, we used Cochrane Library and PubMed medical subject heading (MeSH) on-demand tools to identify alternative terms for the keywords. We searched MEDLINE®, Embase® and Scopus databases from their inception to 6 September 2020. A separate search strategy was developed for each database (Box 1). The search was repeated on 8 February 2022 to identify new articles, and we included additional studies suggested by experts.

Box 1 **Search strategies, systematic review of verbal autopsies in low- and middle-income countries, 1992–2022**

**Medline (Ovid)**

(Records retrieved: 176 on 6 September 2020 and 194 on 8 February 2022)

#1. verbal autops*.mp.

#2. stroke*.mp.

#3. cardio*.mp.

#4. cardia*.mp

#5. isch?em*.mp.

#6. coronary.mp.

#7. angina.mp.

#8. ventric*.mp.

#9. myocard*.mp.

#10. cerebrovasc*.mp.

#11. heart*.mp.

#12. hypertensi*.mp.

#13 (#2 OR #3 OR #4 OR #5 OR #6 OR #7 OR #8 OR #9 OR #10 OR #11 OR #12)

#1 AND #13

**Embase**

(Records retrieved: 273 on 6 September 2020 and 306 on 8 February 2022)

#1. verbal autops*.mp.

#2. stroke*.mp.

#3. cardio*.mp.

#4. cardia*.mp

#5. isch?em*.mp.

#6. coronary.mp.

#7. angina.mp.

#8. ventric*.mp.

#9. myocard*.mp.

#10. cerebrovasc*.mp.

#11. heart*.mp.

#12. hypertensi*.mp.

#13 (stroke* or cardio* or cardia* or isch?em* or coronary or angina or ventric* or myocard* or cerebrovasc* or heart* or hypertensi*).mp.

#1 AND #13

**Scopus**

(Records retrieved: 227 on 6 September 2020 and 248 on 8 February 2022)

#1 (TITLE-ABS-KEY (“verbal autops*”))

#2 (TITLE-ABS-KEY (“stroke*” or cardio* or cardia* or isch?em* or coronary or angina or ventric* or myocard* or cerebrovasc* or heart* or hypertensi*))

# 1 AND #2

### Study selection and data extraction

We used Covidence software (Covidence, Melbourne, Australia) to remove duplicate studies and manage the systematic review. Two reviewers screened titles and abstracts independently, with a third reviewer resolving any conflicts. After the full-text review, a data extraction form was developed and pre-tested on the first five studies identified by each of the two reviewers independently. After comparing the pre-testing results, the form was revised on the basis of consensus findings. Then, the two reviewers independently extracted data from all studies eligible for inclusion in the systematic review. Their findings were compared and any discrepancies were resolved by consensus and with the help of a third reviewer. 

From eligible studies, we extracted data on: (i) the study location; (ii) the study period; (iii) the type of study; (iv) the method of sample selection; (v) the verbal autopsy method used to ascertain the cause of death; (vi) whether the questionnaire was translated; (vii) the recall period for the interview; (viii) the characteristics of data collectors; (ix) the response rate; (x) the total number of verbal autopsy interviews; (xi) the number of deaths due to cardiovascular disease, stroke, ischaemic heart disease, and another or unspecified cardiac disease; (xii) whether deaths were reported by sex or age group; and (xiii) study limitations.

### Risk of bias

We assessed both the external and internal validity of each study included, and data quality was assessed from three broad perspectives using a pre-tested, risk-of-bias assessment tool: (i) selection of study population; (ii) non-response bias; and (iii) measurement bias.^16^ We used six original items from the checklist of this tool (items 1 to 6) and four modified items from the checklist (items 7 to 10) based on our research questions. The resulting 10 items used to assess study bias were: (i) how well the study sample represented the national population; (ii) how well the study sampling frame corresponded to the target population; (iii) the sample selection process; (iv) the response rate; (v) case definitions; (vi) use of a validated questionnaire; (vii) the method used to ascertain the cause of death; (viii) the recall period; (ix) translation of the assessment tools; and (x) training of data collectors. Each item was assessed as having a high or low risk of bias and, in general, an item was categorized as high risk if the study provided unclear or insufficient information. No study was excluded from the review on the basis of its quality. Two reviewers conducted independent risk-of-bias assessments. Thereafter, their findings were compared and any discrepancies were resolved by consensus and with the help of a third reviewer.

### Summary measures

Low- and middle-income countries were identified using the World Bank’s classification for 2019 to 2020.^17^ Cardiovascular diseases were defined using WHO’s 2016 verbal autopsy list and the *International statistical classification of diseases and related health problems, 10th revision*.^9^^,^^18^ The total number of cardiovascular disease deaths was calculated by summing the numbers of deaths from stroke, ischaemic heart disease and other cardiac diseases. The same method was used to calculate cardiovascular disease deaths by sex and age. We used consistent age ranges for all studies to derive age-based distributions. Data are presented as numbers and percentages.

The cause-specific mortality fraction (hereafter mortality fraction) was used to quantify the percentage of deaths in a population due to a particular cause. For each study, we calculated separate mortality fractions for all cardiovascular diseases, stroke, ischaemic heart disease and other cardiac diseases in individuals aged 15 years and above. For different age groups, the cardiovascular disease cause-specific mortality fraction was calculated as the total number of cardiovascular disease deaths in that age group divided by the total number of deaths reported by verbal autopsy in the same age group. We also calculated mortality fractions for these conditions for each sex. Low- and middle-income countries were grouped together into WHO regions. To calculate regional mortality fractions, we added all cardiovascular disease deaths and verbal autopsy deaths, respectively, reported by countries in the same WHO region. Regional mortality fractions for stroke, ischaemic heart disease and other cardiac diseases were calculated using the same method.

## Results

**Fig. 1:**
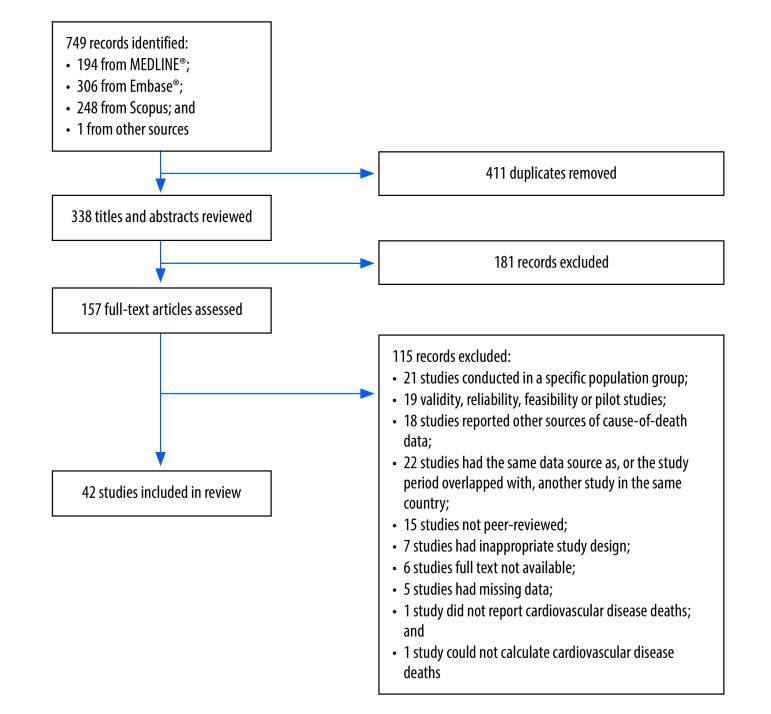
Study selection, systematic review of verbal autopsies in low- and middle-income countries, 1992–2022

In total, 749 studies were identified from the database search and experts’ suggestions. After 411 duplicate publications were removed, the titles and abstracts of 338 studies were screened, 157 studies underwent full-text review and 42 were finally included in the systematic review (Fig. 1).

### Study characteristics

The verbal autopsy data collection period of the studies included in the review ranged from 1992 to 2020 (Table 1).^36^^,^^58^ More than half the studies (24/42) were published between 2000 and 2015.^19^^,^^21^^,^^23^^,^^26^^,^^28^^–^^30^^,^^32^^,^^34^^,^^36^^,^^37^^,^^41^^–^^44^^,^^46^^,^^47^^,^^49^^,^^51^^,^^54^^–^^56^^,^^59^^,^^60^ Studies came from 20 low- and middle-income countries, and covered all WHO regions except for the Region of the Americas. Twenty-two studies were conducted in the African Region,^19^^–^^40^ compared with 13 in the South-East Asia Region,^41^^–^^53^ five in the Western Pacific Region,^54^^–^^58^ one in the Eastern Mediterranean Region,^59^ and one in the European Region.^60^ More than three quarters of the studies (32/42) were surveillance studies.^19^^,^^21^^–^^24^^,^^26^^,^^28^^–^^44^^,^^48^^–^^52^^,^^54^^,^^55^^,^^57^^,^^58^ Of 39 studies that recorded the study setting,^19^^–^^39^^,^^41^^–^^55^^,^^57^^,^^59^^,^^60^ 18 covered rural populations,^26^^,^^28^^,^^30^^,^^32^^,^^34^^,^^35^^,^^37^^,^^39^^,^^41^^–^^44^^,^^48^^–^^50^^,^^52^^,^^53^^,^^55^ six covered urban populations,^21^^,^^25^^,^^46^^,^^47^^,^^51^^,^^59^ and 15 covered both rural and urban populations at the country level.^19^^,^^20^^,^^22^^–^^24^^,^^27^^,^^29^^,^^31^^,^^33^^,^^36^^,^^38^^,^^45^^,^^54^^,^^57^^,^^60^ The number of verbal autopsy deaths reported across all ages ranged between studies from 515 to 22 535,^47^^,^^53^ and 20 studies reported deaths by sex.^19^^,^^23^^,^^26^^,^^28^^,^^31^^,^^33^^,^^35^^,^^42^^–^^44^^,^^47^^,^^48^^,^^50^^–^^52^^,^^54^^–^^56^^,^^58^^,^^60^. Thirty-two studies reported the number of verbal autopsy deaths in people aged 15 years and above; this number ranged from 300 to 472 113.^45^^,^^59^

**Table 1:** Study characteristics, systematic review of verbal autopsies in low- and middle-income countries, 1992–2022

Study^a^	Country	Study setting	Verbal autopsy period	Study design		No. deaths recorded by verbal autopsy						
						All age groups				People aged ≥ 15 years		
						Total^b^	Male	Female		Total^b^	Male	Female
**African Region (*n* = 22) **												
Ndila et al., 2014^19^	Kenya	Urban and rural	2008–2011	Surveillance		4 460	2304	2156		3 310	ND	ND
Chisumpa et al., 2019^20^	Zambia	Urban and rural	2010–2012	Cross-sectional		ND	ND	ND		1 078	582	496
Soura et al., 2014^21^	Burkina Faso	Urban	2009–2011	Surveillance		870	ND	ND		ND	ND	ND
Ashenafi et al., 2017^22^	Ethiopia	Urban and rural	2008–2013	Surveillance		ND	ND	ND		1 535	855	680
Jasseh et al., 2014^23^	Gambia	Urban and rural	1998–2007	Surveillance		2 275	1217	1058		1 619	ND	ND
Abera et al., 2017^24^	Ethiopia	Urban and rural	2009–2015	Surveillance		ND	ND	ND		1 091	547	544
Vusirikala et al., 2019^25^	Kenya	Urban	2008–2018	Cross-sectional		ND	ND	ND		410	ND	ND
Koné et al., 2015^26^	Côte d’Ivoire	Rural	2009–2011	Surveillance		712	386	326		375	218	157
Levira et al., 2019^27^	United Republic of Tanzania	Urban and rural	2011–2014	Cross sectional		5 225	ND	ND		3 257	ND	ND
Mossong et al., 2014^28^	South Africa	Rural	2000–2011	Surveillance		10 958	5140	5818		9 161	ND	ND
Dalinjong et al., 2015^29^	Ghana	Urban and rural	2004–2011	Surveillance		4 021	ND	ND		3 492	2 125	1 367
Kynast-Wolf et al., 2010^30^	Burkina Faso	Rural	1999–2003	Surveillance		1 238	ND	ND		1 238	ND	ND
Rosário et al., 2016^31^	Angola	Urban and rural	2009–2012	Surveillance		934	492	442		407	222	185
Phillips-Howard et al., 2014^32^	Kenya	Rural	2003–2010	Surveillance		ND	ND	ND		15 228	7 295	7 933
Challe et al., 2018^33^	United Republic of Tanzania	Urban and rural	2006–2012	Surveillance		1 325	715	610		713	ND	ND
Awini et al., 2014^34^	Ghana	Rural	2006–2010	Surveillance		3 005	ND	ND		2 547	1 023	1 257
Sifuna et al., 2018^35^	Kenya	Rural	2011–2015	Surveillance		3 903	2063	1840		3 001	1 605	1 396
Walker et al., 2000^36^	United Republic of Tanzania	Urban and rural	1992–1995	Surveillance		11 975	ND	ND		7 629	4 088	3 541
Alabi et al., 2014^37^	Nigeria	Rural	2009–2012	Surveillance		2 050	ND	ND		ND	ND	ND
Natukwatsa et al., 2021^38^	Uganda	Urban and rural	2010–2016	Surveillance		ND	ND	ND		1 210	597	613
Newberry Le Vay et al., 2021^39^	South Africa	Rural	1993–2015	Surveillance		15 305	ND	ND		ND	ND	ND
Fenta et al., 2021^40^	Ethiopia	ND	2007–2017	Surveillance		ND	ND	ND		7 911	4 137	3 774
**South-East Asia Region (*n* = 13)**												
Joshi et al., 2006^41^	India	Rural	2003–2004	Surveillance		1 329	ND	ND		1 251	ND	ND
Alam et al., 2014^42^	Bangladesh	Rural	2003–2010	Surveillance		12 113	6565	5548		9 777	ND	ND
Madhavan et al., 2011^43^	India	Rural	2006–2007	Surveillance		1 827	1007	820		ND	ND	ND
Alam et al., 2014^44^	Bangladesh	Rural	2004–2010	Surveillance		3 231	1759	1472		2 662	ND	ND
Ke et al., 2018^45^	India	Urban and rural	2000–2013	Cross-sectional		ND	ND	ND		472 113	270 000	202 000
Singh et al., 2007^46^	India	Urban	1999–2001	Cross-sectional		ND	ND	ND		2 222	1 385	837
Saha et al., 2007^47^	India	Urban	1994–2004	Cross-sectional		515	340	175		411	ND	ND
Wahab et al., 2017^48^	Indonesia	Rural	2000–2002	Surveillance		830	399	431		775	ND	ND
Rai et al., 2015^49^	India	Rural	2002–2011	Surveillance		ND	ND	ND		4 140	2 508	1 632
Kalkonde et al., 2019^50^	India	Rural	2011–2013	Surveillance		1 599	869	730		1 417	ND	ND
Kanungo et al., 2010^51^	India	Urban	2003–2004	Surveillance		544	322	222		ND	ND	ND
Rai et al., 2020^52^	India	Rural	2012–2017	Surveillance		2 320	1348	972		2 094	1 227	867
Shawon et al., 2021^53^	Bangladesh	Rural	2017–2019	Cross-sectional		22 535	ND	ND		ND	ND	ND
**Western Pacific Region (*n* = 5)**												
Phuong Hoa et al., 2012^54^	Viet Nam	Urban and rural	2008–2009	Surveillance		9 919	5704	4215		9 892	5 700	4 192
Huong et al., 2006^55^	Viet Nam	Rural	1999–2003	Surveillance		1 220	657	563		ND	ND	ND
Ngo et al., 2010^56^	Viet Nam	ND	2006–2007	Cross-sectional		6 798	4078	2727		6 298	3 781	2 517
Gouda et al., 2019^57^	Papua New Guinea	Urban and rural	2009–2014	Surveillance		1 094	ND	ND		ND	ND	ND
Reeve et al., 2021^58^	Solomon Islands	ND	2016–2020	Surveillance		1 034	636	397		ND	ND	ND
**Eastern Mediterranean Region (*n* = 1)**												
Abbas et al., 2011^59^	Pakistan	Urban	2010	Cross-sectional		ND	ND	ND		300	191	109
**European Region (*n* = 1)**												
Akgün et al., 2012^60^	Türkiye	Urban and rural	2002–2004	Cross-sectional		1 089	633	456		ND	ND	ND

ND: not determined.

^a^ We grouped studies by World Health Organization region.

^b^ For some studies, the total number of participants does not equal the sum of male and female participants because of rounding or reporting errors.

### Cardiovascular disease mortality fraction

In total, the 42 studies recorded 129 482 deaths due to cardiovascular disease in individuals aged 15 years and above (Table 2). At the country level, the cardiovascular disease mortality fraction in people aged 15 years or older ranged from 5.5% in Zambia and the United Republic of Tanzania to 63.7% in Pakistan.^20^^,^^36^^,^^59^ In just over half the studies (22/42), the cause of death was ascertained by physicians; ^22^^,^^24^^,^^27^^–^^31^^,^^33^^,^^36^^–^^38^^,^^40^^,^^41^^,^^43^^,^^45^^,^^49^^–^^52^^,^^54^^–^^56^^,^^60^ in 15 studies, cardiovascular disease deaths were ascertained using InterVA (Umeå Centre for Global Health Research, Umeå, Sweden) or SmartVA (Institute for Health Metrics and Evaluation, Seattle, USA) software.^19^^,^^21^^,^^23^^,^^25^^,^^26^^,^^32^^,^^34^^,^^35^^,^^39^^,^^42^^,^^44^^,^^48^^,^^53^^,^^57^^,^^58^

**Table 2:** Cause-specific mortality fraction for cardiovascular disease, systematic review of verbal autopsies in low- and middle-income countries, 1992–2022

Study^a^	Study country	No. deaths recorded by verbal autopsy								No. deaths due to cardiovascular disease								CSMF for cardiovascular disease, %							Verbal autopsy method^b^
		All age groups				People aged ≥ 15 years				All age groups				People aged ≥ 15 years				All age groups				People aged ≥ 15 years			
		Total^c^	Male	Female		Total^c^	Male	Female		Total	Male	Female		Total	Male	Female		Total	Male	Female		Total	Male	Female	
**African Region (*n* = 22)**																									
Ndila^19^	Kenya	ND	ND	ND		3 310	ND	ND		ND	ND	ND		544	ND	ND		ND	ND	ND		16.4	ND	ND	InterVA-4 software
Chisumpa^20^	Zambia	ND	ND	ND		1 078	582	496		ND	ND	ND		59	27	32		ND	ND	ND		5.5	4.6	6.5	ND
Soura^21^	Burkina Faso	870	ND	ND		ND	ND	ND		116	ND	ND		ND	ND	ND		13.3	ND	ND		ND	ND	ND	InterVA-4 software
Ashenafi^22^	Ethiopia	ND	ND	ND		1 535	855	680		ND	ND	ND		163	ND	ND		ND	ND	ND		10.6	ND	ND	Physician-certified
Jasseh^23^	Gambia	ND	ND	ND		1 619	ND	ND		ND	ND	ND		189	ND	ND		ND	ND	ND		11.7	ND	ND	InterVA-4 software
Abera^24^	Ethiopia	ND	ND	ND		1 091	547	544		ND	ND	ND		157	76	81		ND	ND	ND		14.4	13.9	14.9	Physician-certified
Vusirikala^25^	Kenya	ND	ND	ND		410	ND	ND		ND	ND	ND		91	41	47		ND	ND	ND		22.2	ND	ND	InterVA-4 software
Koné^26^	Côte d'Ivoire	ND	ND	ND		375	218	157		ND	ND	ND		25	18	7		ND	ND	ND		6.7	8.3	4.5	InterVA-4 software
Levira^27^	United Republic of Tanzania	5 225	ND	ND		ND	ND	ND		86	38	48		ND	ND	ND		1.6	ND	ND		ND	ND	ND	Physician-certified
Mossong^28^	South Africa	ND	ND	ND		9 161	ND	ND		ND	ND	ND		967	ND	ND		ND	ND	ND		10.6	ND	ND	Physician-certified
Dalinjong^29^	Ghana	ND	ND	ND		3 492	2 125	1 367		ND	ND	ND		371	220	151		ND	ND	ND		10.6	10.4	11	Physician-certified
Kynast-Wolf^30^	Burkina Faso	ND	ND	ND		1 238	ND	ND		ND	ND	ND		113	ND	ND		ND	ND	ND		9.1	ND	ND	Physician-certified
Rosário^31^	Angola	ND	ND	ND		407	222	185		ND	ND	ND		59	24	35		ND	ND	ND		14.5	10.8	18.9	Physician-certified
Phillips-Howard^32^	Kenya	ND	ND	ND		15 228	7 295	7 933		ND	ND	ND		1384	595	789		ND	ND	ND		9.1	8.2	9.9	InterVA-4 software
Challe^33^	United Republic of Tanzania	ND	ND	ND		713	ND	ND		ND	ND	ND		112	ND	ND		ND	ND	ND		15.7	ND	ND	Physician-certified
Awini^34^	Ghana	ND	ND	ND		2 547	1 023	1 257		ND	ND	ND		419	176	243		ND	ND	ND		16.5	17.2	19.5	InterVA-4 software
Sifuna^35^	Kenya	ND	ND	ND		3 001	1 605	1 396		ND	ND	ND		397	ND	ND		ND	ND	ND		13.2	ND	ND	InterVA-4 software
Walker^36^	United Republic of Tanzania	ND	ND	ND		7 629	4 088	3 541		ND	ND	ND		421	225	196		ND	ND	ND		5.5	5.5	5.5	Physician-certified
Alabi^37^	Nigeria	2 050	ND	ND		ND	ND	ND		17	ND	ND		ND	ND	ND		0.8	ND	ND		ND	ND	ND	Physician-certified
Natukwatsa^38^	Uganda	ND	ND	ND		1 210	597	613		ND	ND	ND		260	ND	ND		ND	ND	ND		21.5	ND	ND	Physician-certified
Newberry^39^	South Africa	15 305	ND	ND		ND	ND	ND		1 434	ND	ND		ND	ND	ND		9.4	ND	ND		ND	ND	ND	InterVA-5 software
Fenta^40^	Ethiopia	ND	ND	ND		7 911	4 137	3 774		ND	ND	ND		2 149	1 097	1 052		ND	ND	ND		27.2	26.5	27.9	Physician-certified
Total	NA	23 450	ND	ND		61 955	ND	ND		1 653	ND	ND		7 880	ND	ND		7.0	ND	ND		12.7	ND	ND	NA
South-East Asia Region (*n* = 13)																									
Joshi^41^	India	ND	ND	ND		1 251	ND	ND		ND	ND	ND		431	229	202		ND	ND	ND		34.5	ND	ND	Physician-certified
Alam^42^	Bangladesh	ND	ND	ND		9 777	ND	ND		ND	ND	ND		3 008	1 547	1 461		ND	ND	ND		30.8	ND	ND	InterVA-4 software
Madhavan^43^	India	1 827	1007	820		ND	ND	ND		553	ND	ND		ND	ND	ND		30.3	ND	ND		ND	ND	ND	Physician-certified
Alam^44^	Bangladesh	ND	ND	ND		2 662	ND	ND		ND	ND	ND		903	ND	ND		ND	ND	ND		33.9	ND	ND	InterVA-4 software
Ke^45^	India	ND	ND	ND		472 113	270 000	202 000		ND	ND	ND		111 977	68 904	43 073		ND	ND	ND		23.7	25.5	21.3	Physician-certified
Singh^46^	India	ND	ND	ND		2 222	1 385	837		ND	ND	ND		646	406	240		ND	ND	ND		29.1	29.3	27.4	ND
Saha^47^	India	ND	ND	ND		411	ND	ND		ND	ND	ND		42	26	16		ND	ND	ND		10.2	ND	ND	Medical officer-certified
Wahab^48^	Indonesia	ND	ND	ND		775	ND	ND		ND	ND	ND		318	ND	ND		ND	ND	ND		41	ND	ND	InterVA-4 software
Rai^49^	India	ND	ND	ND		4 140	2 508	1 632		ND	ND	ND		1 413	895	518		ND	ND	ND		34.1	35.7	31.7	Physician-certified
Kalkonde^50^	India	ND	ND	ND		1 417	ND	ND		ND	ND	ND		332	175	157		ND	ND	ND		23.4	ND	ND	Physician-certified
Kanungo^51^	India	544	322	222		ND	ND	ND		198	106	92		ND	ND	ND		36.4	32.9	41.4		ND	ND	ND	Physician-certified
Rai^52^	India	ND	ND	ND		2 094	1227	867		ND	ND	ND		685	358	327		ND	ND	ND		32.7	29.2	37.7	Physician-certified
Shawon^53^	Bangladesh	22 535	ND	ND		ND	ND	ND		9 331	5 759	3 572		ND	ND	ND		41.4	ND	ND		ND	ND	ND	SmartVA software
Total	NA	24 906	ND	ND		496 862	ND	ND		10 082	ND	ND		119 755	ND	ND		40.5	ND	ND		24.1	ND	ND	NA
Western Pacific Region (*n* = 5)																									
Phuong Hoa^54^	Viet Nam	9 919	5704	4215		ND	ND	ND		629	209	420		ND	ND	ND		6.3	3.7	10.0		ND	ND	ND	Physician-certified
Huong^55^	Viet Nam	1 220	657	563		ND	ND	ND		353	193	160		ND	ND	ND		28.9	29.4	28.4		ND	ND	ND	Physician-certified
Ngo^56^	Viet Nam	6 798	4078	2727		6 298	3 781	2 517		ND	ND	ND		1 656	884	772		ND	ND	ND		26.3	23.4	30.7	Physician-certified
Gouda^57^	Papua New Guinea	1 094	ND	ND		ND	ND	ND		69	38	31		ND	ND	ND		6.3	ND	ND		ND	ND	ND	SmartVA software
Reeve^58^	Solomon Islands	1 034	636	397		ND	ND	ND		281	195	86		ND	ND	ND		27.2	30.7	21.7		ND	ND	ND	SmartVA software
Total	NA	13 267	ND	ND		6 298	ND	ND		1 332	ND	ND		1 656	ND	ND		10.0	ND	ND		26.3	ND	ND	NA
**Eastern Mediterranean Region (*n* = 1)**																									
Abbas^59^	Pakistan	ND	ND	ND		300	191	109		ND	ND	ND		191	ND	ND		ND	ND	ND		63.7	ND	ND	ND
Total	NA	ND	ND	ND		300	ND	ND		ND	ND	ND		191	ND	ND		ND	ND	ND		63.7	ND	ND	NA
**European Region (*n* = 1)**																									
Akgün^60^	Türkiye	1 089	633	456		ND	ND	ND		314	183	131		ND	ND	ND		28.8	28.9	28.7		ND	ND	ND	Physician-certified
Total	NA	1 089	ND	ND		ND	ND	ND		314	ND	ND		ND	ND	ND		28.8	ND	ND		ND	ND	ND	NA
Total for all regions	NA	62 712	ND	ND		565 415	ND	ND		13 381	ND	ND		129 482	ND	ND		21.3	ND	ND		**22.9**	**ND**	**ND**	**NA**

CSMF: cause-specific mortality fraction; NA: not applicable; ND: not determined.

^a^ Countries were grouped by World Health Organization region.

^b^ The results of verbal autopsies were either certified by a physician or medical officer or coded using a data-driven computer algorithm, such as InterVA or SmartVA.

^c^ For some studies, the total number of participants does not equal the sum of male and female participants because of rounding or reporting errors.

Overall, the cardiovascular disease mortality fraction was 21.3% across all age groups and 22.9% in people aged 15 years or older (Table 2). By WHO region, the cardiovascular disease mortality fraction in people aged 15 years or older was 26.3% in the Western Pacific Region; 24.1% in the South-East Asia Region; and 12.7% in the African Region. 

Fourteen studies reported both cardiovascular disease deaths by sex and verbal autopsy deaths in people aged 15 years or older (Table 2).^20^^,^^24^^,^^26^^,^^29^^,^^31^^,^^32^^,^^34^^,^^36^^,^^40^^,^^45^^,^^46^^,^^49^^,^^52^^,^^56^ Overall, the cardiovascular disease mortality fraction was higher in males than females: 24.7% versus 20.9%, respectively (Table 3). Although the pattern was similar in the South-East Asia Region, the cardiovascular disease mortality fraction was higher in females than males in the African and Western Pacific Regions.

**Table 3:** Cause-specific mortality fraction for cardiovascular disease, by sex and WHO region, systematic review of verbal autopsies in low- and middle-income countries, 1992–2022

WHO region	No. studies	Parameter for people aged ≥ 15 years										
		No. deaths recorded by verbal autopsy				No. deaths due to cardiovascular disease				Cause-specific mortality fraction for cardiovascular disease, %		
		Total^a^	Male	Female		Total	Male	Female		Total	Male	Female
African^20^^,^^24^^,^^26^^,^^29^^,^^31^^,^^32^^,^^34^^,^^36^^,^^40^	9	39 758	20 237	19 254		5 044	2 458	2 586		12.7	12.1	13.4
South-East Asia^45^^,^^46^^,^^49^^,^^52^	4	480 569	275 120	205 374		114 721	70 563	44 158		23.9	25.6	21.5
Western Pacific^56^	1	6 298	3 781	2 517		1 656	884	772		26.3	23.4	30.7
Total	14	526 625	299 138	227 145		121 421	73 905	47 516		23.1	24.7	20.9

WHO: World Health Organization.

^a^ For some regions, the total number of participants does not equal the sum of male and female participants because of rounding or reporting errors in individual studies.

### Study setting

Sixteen studies reported the number of verbal autopsy deaths and the number of cardiovascular disease deaths in people aged 15 years or older by rural or urban residence: 13 were performed in rural areas and three were performed in urban areas (Table 4).^25^^,^^26^^,^^28^^,^^30^^,^^32^^,^^34^^,^^35^^,^^41^^,^^42^^,^^44^^,^^46^^–^^50^^,^^52^ Overall, the cardiovascular disease mortality fraction was higher in urban than in rural settings: 25.6% versus19.4%, respectively. In the African Region, the cardiovascular disease mortality fraction was higher in urban than rural populations (22.2% versus 10.5%, respectively), whereas in the South-East Asia Region it was higher in rural than urban populations (32.1% versus 26.1%, respectively).

**Table 4:** Cause-specific mortality fraction for cardiovascular disease, by study setting and WHO region, systematic review of verbal autopsies in low- and middle-income countries, 1992–2022

Study setting and WHO region	No. studies		Parameter for people aged ≥ 15 years		
			No. deaths recorded by verbal autopsy	No. deaths due to cardiovascular disease	Cause-specific mortality fraction for cardiovascular disease, %
**Rural**					
African^26^^,^^28^^,^^30^^,^^32^^,^^34^^,^^35^	6		31 550	3 305	10.5
South-East Asia^41^^,^^42^^,^^44^^,^^48^^–^^50^^,^^52^	7		22 116	7 090	32.1
Total	13		53 666	10 395	19.4
**Urban**					
African^25^	1		410	91	22.2
South-East Asia^46^^,^^47^	2		2 633	688	26.1
Total	3		3 043	779	25.6

WHO: World Health Organization.

### Differences by age

Seven studies reported cardiovascular disease deaths in the age groups 15 to 49 years, 50 to 64 years and 65 years or older (Table 5).^19^^,^^26^^,^^28^^,^^34^^,^^35^^,^^42^^,^^44^ In these studies, 69.4% of cardiovascular disease deaths were reported in people aged 65 years or older, and 20.2% were reported in people aged 50 to 64 years. Six studies reported cardiovascular disease deaths in the age groups 15 to 59 years and 60 years or older (Table 6).^23^^,^^33^^,^^41^^,^^49^^,^^50^^,^^56^ Among these studies, 80.5% of cardiovascular disease deaths were reported in people aged 60 years or older.

**Table 5:** Cardiovascular disease deaths, by age group (15–49 years, 50–64 years and ≥ 65 years), systematic review of verbal autopsies in low- and middle-income countries, 1992–2022

Study author, country	Cardiovascular disease deaths			
	All age groups^a^	15–49 years	50–64 years	≥ 65 years
Alam, Bangladesh^42^	3008	242	559	2167
Ndila, Kenya^19^	544	64	116	364
Koné, Côte d’Ivoire^26^	25	4	6	15
Mossong, South Africa^28^	969	103	230	634
Alam, Bangladesh^44^	903	86	185	632
Awini, Ghana^34^	419	53	104	262
Sifuna, Kenya^35^	398	59	66	272
**Total (%)**	**6266 (100)**	**611 (9.8)**	**1266 (20.2)**	**4346 (69.4)**

^a^ For some studies, the number for all age groups also included individuals aged under 15 years.

**Table 6:** Cardiovascular disease deaths, by age group (15–59 years and ≥ 60 years), systematic review of verbal autopsies in low- and middle-income countries, 1992–2022

Study author, country	Cardiovascular disease deaths		
	All age groups^a^	15–59 years	≥ 60 years
Joshi, India^41^	431	124	310
Jasseh, Gambia^23^	196	44	145
Ngo, Viet Nam^56^	1656	201	1455
Kalkonde, India^50^	332	100	232
Challe, United Republic of Tanzania^33^	112	11	101
Rai, India^49^	685	182	502
**Total (%)**	**3412 (100)**	**663 (19.4)**	**2745 (80.5)**

^a^ For some studies, the number for all age groups also included individuals aged under 15 years.

### Type of cardiovascular disease

Overall in people aged 15 years or older, the mortality fraction for ischaemic heart disease (12.3%) was higher than that for stroke (8.7%) and for other or unspecified heart disease (1.5%; Table 7). The pattern was similar in the South-East Asia Region. In the African Region, however, the mortality fraction for stroke (4.2%) was higher than that for ischaemic heart disease (0.8%).

**Table 7:** Cause-specific mortality fraction, by type of cardiovascular disease, systematic review of verbal autopsies in low- and middle-income countries, 1992–2022

Study author, country^a^	Verbal autopsy findings in people aged ≥ 15 years									
	Total deaths (*n*)		Stroke			Ischaemic heart disease			Other and unspecified cardiac disease	
			No. deaths	Cause-specific mortality fraction, %		No. deaths	Cause-specific mortality fraction, %		No. deaths	Cause-specific mortality fraction, %
**African Region (*n* = 13)**										
Ndila, Kenya^19^	3 310		317	9.6		33	1.0		194	5.9
Ashenafi, Ethiopia^22^	1 535		64	4.2		30	2.0		69	4.5
Jasseh, Gambia^23^	1 619		146	9.0		ND	ND		43	2.7
Abera, Ethiopia^24^	1 091		83	7.6		26	2.4		48	4.4
Koné, Côte d’Ivoire^26^	375		9	2.4		1	0.3		15	4.0
Mossong, South Africa^28^	9 161		403	4.4		55	0.6		509	5.6
Kynast-Wolf, Burkina Faso^30^	1 238		15	1.2		ND	ND		ND	ND
Phillips-Howard, Kenya^32^	15 228		327	2.1		100	0.7		957	6.3
Challe, United Republic of Tanzania^33^	713		41	5.8		ND	ND		71	10.0
Awini, Ghana^34^	2 547		219	8.6		147	5.8		53	2.1
Sifuna, Kenya^35^	3 001		201	6.7		74	2.5		122	4.1
Walker, United Republic of Tanzania^36^	7 629		421	5.5		ND	ND		ND	ND
Fenta, Ethiopia^40^	7 911		81	1.0		ND	ND		155	2.0
Total	55 358		2327	4.2		466	0.8		2236	4.0
**South-East Asia Region (*n* = 10)**										
Joshi, India^41^	1 251		170	13.6		183	14.6		78	6.2
Alam, Bangladesh^44^	9 777		2144	21.9		863	8.8		ND	ND
Alam, Bangladesh^42^	2 662		569	21.4		335	12.6		ND	ND
Ke, India^45^	472 113		41 000	8.7		66 000	14.0		5000	1.1
Singh, India^46^	2 222		175	7.9		267	12.0		204	9.2
Saha, India^47^	411		ND	ND		42	10.2		42	10.2
Wahab, Indonesia^48^	775		213	27.5		9	1.2		96	12.4
Rai, India^52^	4 140		122	2.9		426	10.3		53	1.3
Kalkonde, India^50^	1 417		229	16.2		69	4.9		7	0.5
Rai, India^52^	2 094		558	26.6		91	4.3		33	1.6
Total	496 862		45 180	9.1		68 285	13.7		5513	1.1
**Western Pacific Region (*n* = 1)**										
Ngo, Viet Nam^56^	6 298		1139	18.1		136	2.2		381	6.0
Total	6 298		1139	18.1		136	2.2		381	6.0
**Total for all regions**	558 518		48 646	8.7		68 887	12.3		8130	1.5

ND: not determined.

^a^ We grouped countries by World Health Organization regions.

### Risk of bias 

**Fig. 2:**
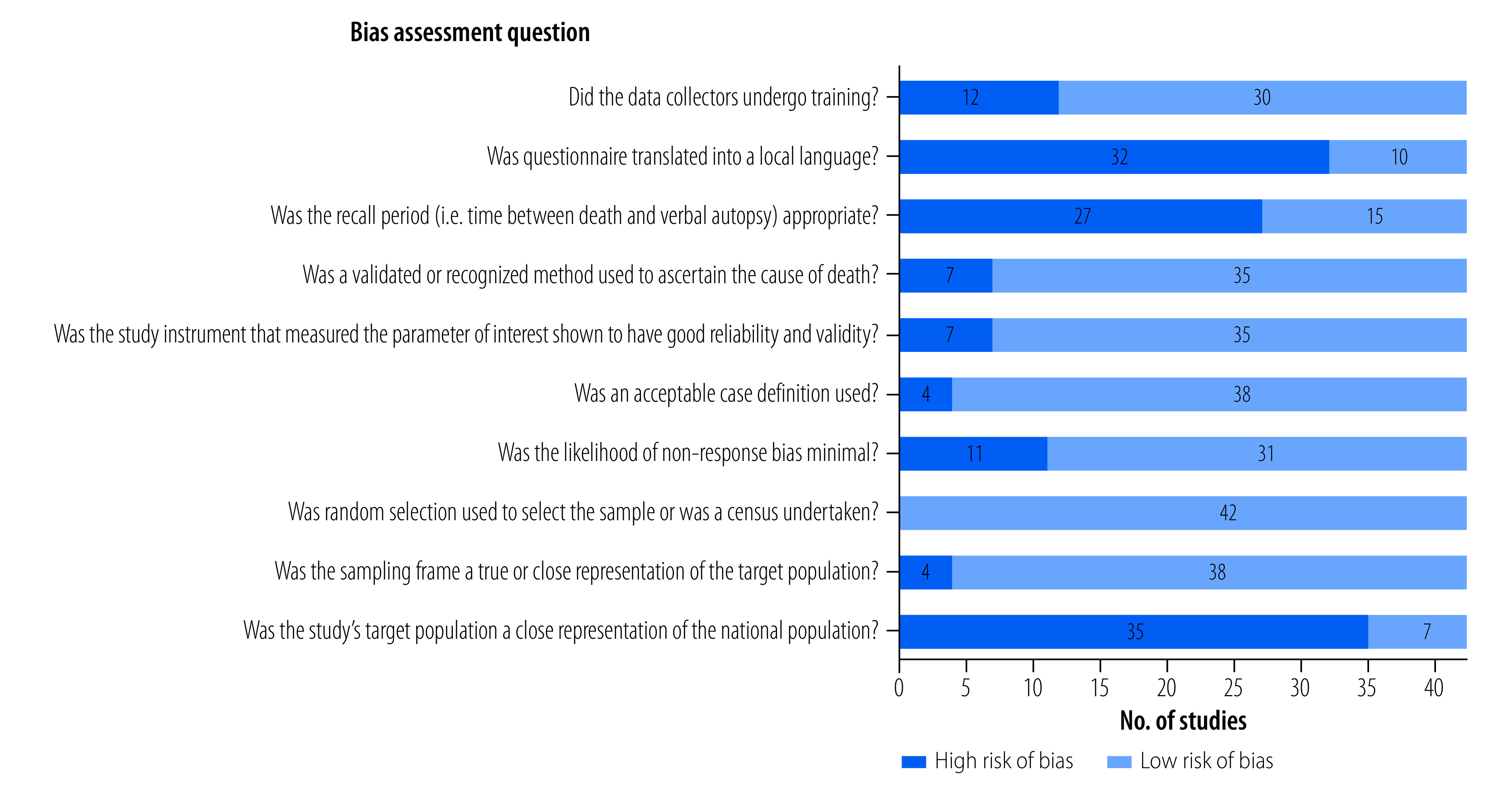
Risk-of-bias assessment, systematic review of verbal autopsies in low- and middle-income countries, 1992–2022

The findings of the risk-of-bias assessments in the 42 studies are shown in Fig. 2. Overall, 83% (35/42) of studies had poorly reported or unclear information on how representative the study target population was of the national population. Moreover, 76% (32/42) of studies did not report whether the verbal autopsy questionnaire had been translated into a local language. Information on whether the recall period between the person’s death and the verbal autopsy was appropriate (i.e. under 3 months) was either absent or unclear in 64% (27/42) of studies. Full details of the risk-of-bias assessments for individual studies are available from the data repository.^12^

## Discussion

We found that the overall cardiovascular disease mortality fraction among people in low- and middle-income countries aged 15 years or older was 22.9%, and that the mortality fraction was generally higher in males than females. Moreover, the mortality fraction varied with age, geographical location and the type of cardiovascular disease. The highest burden of cardiovascular disease deaths was reported in WHO’s Western Pacific Region, followed by the South-East Asia Region and the African Region. The cardiovascular disease mortality fraction was higher in urban than rural populations in all regions except the South-East Asia Region. We also found that the mortality fraction was generally higher for ischaemic heart disease than stroke, though stroke deaths were more common in Africa.

Verbal autopsy is an important data source for the GBD, which produces global, regional and national estimates of the frequency of different causes of death.^1^ Our review provides new data on cardiovascular disease mortality from published verbal autopsy studies that may not previously have been included in GBD estimates, and which could increase the representativeness of global estimates. Moreover, our review provides data on rural and urban populations and on regions where information on cardiovascular disease mortality is scarce because there is no adequate death registration system. The inclusion of verbal autopsy data from regions and population groups that are underrepresented in existing global estimates will help make estimates for these regions more balanced and accurate. Although our review did not include data from the WHO Region of the Americas, verbal autopsy is not needed in most of the region because the cause of death is recorded by medical certification, except in some very remote communities where verbal autopsy is used (e.g. in Colombia).^61^

Although our findings may not be generalizable to a global or national level, a comparison with GBD estimates is helpful. Our overall estimate of the cardiovascular disease mortality fraction of 22.9% is lower than that estimated by the 2019 GBD study (the most recent), which found a cardiovascular disease mortality fraction of 32% across all age groups globally.^1^ In addition, our review found a higher cardiovascular disease mortality fraction in males than females overall, which was not in agreement with the 2019 GBD estimates.^1^ Nevertheless, the regional sex differences in cardiovascular disease mortality fraction we found in our review were consistent with GBD estimates.^1^ Our observations that the mortality fraction for ischaemic heart disease was higher than that for stroke, and that the cardiovascular disease mortality fraction was higher in older than younger age groups, were similar to GBD findings.^1^

The differences between our review’s findings and GBD estimates could be due to the lack of generalizability of our study data. Our review included few studies from the Western Pacific, Eastern Mediterranean or European Regions, or from high-income countries where death due to cardiovascular disease is more common.^1^ In addition, the studies included in our review mainly focused on deaths at home, which are most frequently assessed by verbal autopsy. By contrast, the GBD estimates mortality fractions for all deaths in all countries and regions.^2^ Moreover, GBD estimates of the global cardiovascular disease mortality fraction are affected by a lack of data from some countries, notably countries with a high proportion of deaths in the community, such as those in sub-Saharan Africa and South-East Asia,^3^ which may help explain why our cardiovascular disease mortality fraction estimates were lower. Our review suggests that the verbal autopsy method can help fill gaps in cardiovascular disease mortality data for low- and middle-income countries that do not have adequate vital registration systems, and can be a valuable tool for identifying different types of cardiovascular death in the community.

Most studies (32/42) in our review were surveillance studies and did not report whether the study population was comparable with the national population in terms of age, sex, socioeconomic status or any other factor. Surveillance studies would be more valuable if they reported the characteristics of the study population, which, in turn, would help establish the generalizability of the study’s findings. Moreover, to minimize assessment errors, studies should report whether the verbal autopsy questionnaire has been translated into a local language, and the time delay between death and the autopsy interview; the diagnosis is more likely to be correct if the time delay is short.^8^

Our systematic review had several limitations. First, the number of studies included varied considerably between regions. In addition, the studies included diverse population groups and involved different autopsy methods. The resulting heterogeneity between the studies may limit the generalizability and comparability of our findings at regional and country levels. Second, our review calculated the cardiovascular disease mortality fraction only for individuals aged 15 years or older, because most studies included in the review reported cardiovascular disease mortality in that age range and not in younger age groups. Although focusing on older individuals provides valuable insights into the prevalence of death due to cardiovascular disease, including younger individuals would have helped identify emerging trends and assisted public health planning. Furthermore, the variation in age group categories between studies limited our ability to achieve a complete understanding of cardiovascular mortality across all age groups. Verbal autopsy studies should publish their results in a greater number of age groups, as this would enable the influence of age on cardiovascular disease mortality to be better investigated. Third, as mentioned, the generalizability of our study results was limited because most studies included were surveillance studies conducted in one specific geographical area, and most considered deaths occurring outside of a health-care setting. The use of a standardized assessment tool and cross-validation with other national and international data would increase the generalizability of verbal autopsy study findings to other populations.^9^ Fourth, as we only calculated the cardiovascular disease mortality fraction for verbal autopsy deaths and not for all deaths, the mortality fraction is likely to differ from that derived from deaths in hospital or other locations. Finally, this systematic review included all data irrespective of when they had been collected. Although including only recent studies would have provided the most up-to-date data on cardiovascular mortality, we wanted our review to include as many large studies as possible. As the mortality fraction for cardiovascular disease has been increasing in low- and middle-income countries, the use of more recent data would likely have produced a higher mortality fraction. Moreover, newer studies may have used improved data collection methods and have been better at attributing the cause of death to cardiovascular disease. For example, computer-coded verbal autopsy has become more popular and has been shown to be more accurate for confirming death due to heart disease than physician-certified verbal autopsy.^62^

The verbal autopsy method also has limitations. The consistency of the symptoms reported by relatives during the verbal autopsy interview has been reported as low, especially when interviews take place a long time after the death.^63^ Nevertheless, despite the low consistency, reported symptoms were generally sufficient for assigning the cause of death,^63^ which is important given that verbal autopsy is only source of information about the cause of death at the population level in many low- and middle-income countries.^64^ Future studies involving verbal autopsies should focus on minimizing recall bias by using validated questionnaires, and should ensure interviews take place within 3 months of the mourning period.^8^ The studies in our review used different methods to ascertain the cause of death, with nearly half using the physician-certified method. A previous systematic review showed that physician-certified verbal autopsy was relatively poor at confirming heart disease compared with computer-coded verbal autopsy, though it was based on only three studies of hospital deaths.^65^ More data are needed to understand the performance of different verbal autopsy methods in confirming different types of death, especially death at home. Verbal autopsy findings are specific to the population or setting in which the autopsies are conducted and it is, therefore, difficult to generalize them to other contexts. Recently, however, verbal autopsy has become routine in some settings. In particular, it has become part of civil registration and vital statistics systems in countries such as Bangladesh.^53^ As a result, data on deaths due to cardiovascular disease and other causes will become more generalizable. Future studies using these data could validate verbal autopsy findings across diverse populations and geographical areas.

In many settings, the quality of verbal autopsy data directly affects health policy. A systematic review of 66 validation studies of verbal autopsy published in 2022 compared the cause of death assigned by verbal autopsy to the cause of death assigned by other methods such as autopsy diagnosis and hospital diagnosis.^66^ The review found that the majority of studies reported an acceptable level of agreement between verbal autopsy and the comparison method as assessed, using measures such as chance-corrected concordance, kappa coefficients, sensitivity, specificity or the positive predictive value. Although the review confirmed the validity of verbal autopsy methods, it also highlighted gaps in the quality of verbal autopsy studies involving, for example, the use of non-validated questionnaires; the time delay between death and the verbal autopsy interview; and problems with the cause-of-death assignment technique.^66^

In conclusion, our systematic review provides evidence that the burden of cardiovascular disease deaths outside health-care settings is substantial. More data and research are needed to gain a better understanding of whether variations in the cardiovascular disease mortality fraction for community deaths across regions, subnational populations and sexes are indicative of health inequalities. Future verbal autopsy studies examining cardiovascular disease mortality should be more representative of the national population and should ensure minimal recall bias. Further investment to increase coverage of verbal autopsies in low- and middle-income countries would help fill gaps in cardiovascular disease mortality data, and improve the monitoring of national, regional and global health goals.

## References

[1] Results from the 2019 Global Burden of Disease (GBD) study. Seattle: Institute for Health Metrics and Evaluation; 2022. Available from: https://ghdx.healthdata.org/gbd-results-tool [cited 2022 Jul 10].

[2] Roth GA, Abate D, Abate KH, Abay SM, Abbafati C, Abbasi N, et al. GBD 2017 Causes of Death Collaborators. Global, regional, and national age-sex-specific mortality for 282 causes of death in 195 countries and territories, 1980–2017: a systematic analysis for the Global Burden of Disease Study 2017. Lancet. 2018 Nov 10;392(10159):1736–88. 10.1016/S0140-6736(18)32203-7 30496103 PMC6227606

[3] Roth GA, Mensah GA, Johnson CO, Addolorato G, Ammirati E, Baddour LM, et al. GBD-NHLBI-JACC Global Burden of Cardiovascular Diseases Writing Group. Global burden of cardiovascular diseases and risk factors, 1990–2019: update from the GBD 2019 Study. J Am Coll Cardiol. 2020 Dec 22;76(25):2982–3021. 10.1016/j.jacc.2020.11.010 33309175 PMC7755038

[4] 3.4. By 2030 reduce by one-third premature mortality from non-communicable diseases (NCDs) through prevention and treatment, and promote mental health and wellbeing. New York: Sustainable Development Solutions Network; 2015. Available from: http://indicators.report/targets/3-4 [cited 2022 Jan 11].

[5] Mikkelsen L, Phillips DE, AbouZahr C, Setel PW, de Savigny D, Lozano R, et al. A global assessment of civil registration and vital statistics systems: monitoring data quality and progress. Lancet. 2015 Oct 3;386(10001):1395–406. 10.1016/S0140-6736(15)60171-4 25971218

[6] AbouZahr C, de Savigny D, Mikkelsen L, Setel PW, Lozano R, Nichols E, et al. Civil registration and vital statistics: progress in the data revolution for counting and accountability. Lancet. 2015 Oct 3;386(10001):1373–85. 10.1016/S0140-6736(15)60173-8 25971224 PMC7753937

[7] Adair T, Firth S, Phyo TPP, Bo KS, Lopez AD. Monitoring progress with national and subnational health goals by integrating verbal autopsy and medically certified cause of death data. BMJ Glob Health. 2021 May;6(5):e005387. 10.1136/bmjgh-2021-005387 34059494 PMC8169488

[8] Serina P, Riley I, Hernandez B, Flaxman AD, Praveen D, Tallo V, et al. What is the optimal recall period for verbal autopsies? Validation study based on repeat interviews in three populations. Popul Health Metr. 2016 Oct 18;14(1):40. 10.1186/s12963-016-0105-1 27833459 PMC5101705

[9] Verbal autopsy standards: the 2022 WHO verbal autopsy instrument VI. Geneva: World Health Organization; 2022. Available from: https://cdn.who.int/media/docs/default-source/classification/other-classifications/autopsy/2022-va-instrument/verbal-autopsy-standards_2022-who-verbal-autopsy-instrument_v1_final.pdf?sfvrsn=c8cf2dda_8 [cited 2023 June 29].

[10] de Savigny D, Riley I, Chandramohan D, Odhiambo F, Nichols E, Notzon S, et al. Integrating community-based verbal autopsy into civil registration and vital statistics (CRVS): system-level considerations. Glob Health Action. 2017;10(1):1272882. 10.1080/16549716.2017.1272882 28137194 PMC5328373

[11] Leitao J, Chandramohan D, Byass P, Jakob R, Bundhamcharoen K, Choprapawon C, et al. Revising the WHO verbal autopsy instrument to facilitate routine cause-of-death monitoring. Glob Health Action. 2013 Sep 13;6(1):21518. 10.3402/gha.v6i0.21518 24041439 PMC3774013

[12] Acharya A, Chowdhury HR, Ihyauddin Z, Mahesh PKB, Adair T. Inclusion and exclusion criteria and risk of bias assessment of individual studies. Cardiovascular disease mortality based on verbal autopsy in low- and middle-income countries: a systematic review. London: Figshare; 2023. 10.26188/23605716 PMC10452938 37638359

[13] Moher D, Liberati A, Tetzlaff J, Altman DG, PRISMA Group. Preferred reporting items for systematic reviews and meta-analyses: the PRISMA statement. Ann Intern Med. 2009;151(4):264-9. https://dx.doi/10.7326/0003-4819-151-4-200908180-00135 10.1136/bmjgh-2017-000639 19622511

[14] Acharya A, Adair T, Chowdhury MH, Koralage BP, Ihyauddin Z. Measuring cardiovascular mortality in low- and middle-income countries: a systematic review of verbal autopsy studies. International prospective register of systematic reviews (PROSPERO). York: Centre for Reviews and Dissemination; 2023. Available from: https://www.crd.york.ac.uk/PROSPERO/display_record.php?RecordID=210768 [cited 2020 Nov 20].

[15] Schardt C, Adams MB, Owens T, Keitz S, Fontelo P. Utilization of the PICO framework to improve searching PubMed for clinical questions. BMC Med Inform Decis Mak. 2007 Jun 15;7(1):16. 10.1186/1472-6947-7-16 17573961 PMC1904193

[16] Hoy D, Brooks P, Woolf A, Blyth F, March L, Bain C, et al. Assessing risk of bias in prevalence studies: modification of an existing tool and evidence of interrater agreement. J Clin Epidemiol. 2012 Sep;65(9):934–9. 10.1016/j.jclinepi.2011.11.014 22742910

[17] World Bank country and lending groups. Country classification by income 2019–2020. Washington, DC: World Bank; 2023. Available from: https://datahelpdesk.worldbank.org/knowledgebase/articles/906519-world-bank-country-and-lending-groups [cited 2022 Nov 15].

[18] International statistical classification of diseases and related health problems, 10th revision. Geneva: World Health Organization; 2010. Available from: https://www.who.int/publications/m/item/international-statistical-classification-of-diseases-and-related-health-problems---volume-2 [cited 2020 Nov 15].

[19] Ndila C, Bauni E, Mochamah G, Nyirongo V, Makazi A, Kosgei P, et al. Causes of death among persons of all ages within the Kilifi Health and Demographic Surveillance System, Kenya, determined from verbal autopsies interpreted using the InterVA-4 model. Glob Health Action. 2014 Oct 29;7(1):25593. 10.3402/gha.v7.25593 25377342 PMC4220144

[20] Chisumpa VH, Odimegwu CO, Saikia N. Adult mortality in sub-Saharan Africa: cross-sectional study of causes of death in Zambia. Trop Med Int Health. 2019 Oct;24(10):1208–20. 10.1111/tmi.13302 31420929

[21] Soura AB, Lankoande B, Millogo R, Bangha M. Comparing causes of death between formal and informal neighborhoods in urban Africa: evidence from Ouagadougou Health and Demographic Surveillance System. Glob Health Action. 2014 Oct 29;7(1):25523. 10.3402/gha.v7.25523 25377335 PMC4220135

[22] Ashenafi W, Eshetu F, Assefa N, Oljira L, Dedefo M, Zelalem D, et al. Trend and causes of adult mortality in Kersa Health and Demographic Surveillance System (Kersa HDSS), eastern Ethiopia: verbal autopsy method. Popul Health Metr. 2017 Jul 1;15(1):22. 10.1186/s12963-017-0144-2 28666480 PMC5493878

[23] Jasseh M, Howie SR, Gomez P, Scott S, Roca A, Cham M, et al. Disease-specific mortality burdens in a rural Gambian population using verbal autopsy, 1998–2007. Glob Health Action. 2014 Oct 29;7(1):25598. 10.3402/gha.v7.25598 25377344 PMC4220164

[24] Abera SF, Gebru AA, Biesalski HK, Ejeta G, Wienke A, Scherbaum V, et al. Social determinants of adult mortality from non-communicable diseases in northern Ethiopia, 2009–2015: evidence from Health and Demographic Surveillance site. PLoS One. 2017 Dec 13;12(12):e0188968. 10.1371/journal.pone.0188968 29236741 PMC5728486

[25] Vusirikala A, Wekesah F, Kyobutungi C, Oyebode O. Assessment of cardiovascular risk in a slum population in Kenya: use of World Health Organization/International Society of Hypertension (WHO/ISH) risk prediction charts – secondary analyses of a household survey. BMJ Open. 2019 Sep 4;9(9):e029304. 10.1136/bmjopen-2019-029304 31488481 PMC6731939

[26] Koné S, Fürst T, Jaeger FN, Esso ELJC, Baïkoro N, Kouadio KA, et al. Causes of death in the Taabo Health and Demographic Surveillance System, Côte d’Ivoire, from 2009 to 2011. Glob Health Action. 2015 May 8;8(1):27271. 10.3402/gha.v8.27271 25959772 PMC4426287

[27] Levira F, Newton CR, Masanja H, Odermatt P. Mortality of neurological disorders in Tanzania: analysis of baseline data from sample vital registration with verbal autopsy (SAVVY). Glob Health Action. 2019;12(1):1596378. 10.1080/16549716.2019.1596378 31144608 PMC7011788

[28] Mossong J, Byass P, Herbst K. Who died of what in rural KwaZulu-Natal, South Africa: a cause of death analysis using InterVA-4. Glob Health Action. 2014 Oct 29;7(1):25496. 10.3402/gha.v7.25496 25377332 PMC4220127

[29] Dalinjong PA, Welaga P, Azongo DK, Chatio S, Anaseba D, Kondayire F, et al. A retrospective analysis of the association between tobacco smoking and deaths from respiratory and cardiovascular diseases in the Kassena-Nankana districts of Northern Ghana. Tob Induc Dis. 2015 Apr 26;13(1):12. 10.1186/s12971-015-0037-8 25937824 PMC4416277

[30] Kynast-Wolf G, Preuß M, Sié A, Kouyaté B, Becher H. Seasonal patterns of cardiovascular disease mortality of adults in Burkina Faso, West Africa. Trop Med Int Health. 2010 Sep;15(9):1082–9. 10.1111/j.1365-3156.2010.02586.x 20667050

[31] Rosário EVN, Costa D, Timóteo L, Rodrigues AA, Varanda J, Nery SV, et al. Main causes of death in Dande, Angola: results from verbal autopsies of deaths occurring during 2009–2012. BMC Public Health. 2016 Aug 4;16(1):719. 10.1186/s12889-016-3365-6 27491865 PMC4973533

[32] Phillips-Howard PA, Laserson KF, Amek N, Beynon CM, Angell SY, Khagayi S, et al. Deaths ascribed to non-communicable diseases among rural Kenyan adults are proportionately increasing: evidence from a Health and Demographic Surveillance System, 2003–2010. PLoS ONE. 2014 Nov 26;9(11):e114010. 10.1111/j.1365-3156.2010.02586.x 25426945 PMC4245262

[33] Challe DP, Kamugisha ML, Mmbando BP, Francis F, Chiduo MG, Mandara CI, et al. Pattern of all-causes and cause-specific mortality in an area with progressively declining malaria burden in Korogwe district, north-eastern Tanzania. Malar J. 2018 Feb 27;17(1):97. 10.1186/s12936-018-2240-6 29482553 PMC5828081

[34] Awini E, Sarpong D, Adjei A, Manyeh AK, Amu A, Akweongo P, et al. Estimating cause of adult (15+ years) death using InterVA-4 in a rural district of southern Ghana. Glob Health Action. 2014 Oct 29;7(1):25543. 10.3402/gha.v7.25543 25377337 PMC4220134

[35] Sifuna P, Otieno L, Ogwang S, Ogutu B, Andagalu B, Owuoth J, et al. Cause-specific mortality in the Kombewa Health and Demographic Surveillance Systems site, rural Western Kenya from 2011–2015. Glob Health Action. 2018;11(1):1442959. 10.1080/16549716.2018.1442959 29502491 PMC5844040

[36] Walker RW, McLarty DG, Kitange HM, Whiting D, Masuki G, Mtasiwa DM, et al. Stroke mortality in urban and rural Tanzania. Adult Morbidity and Mortality Project. Lancet. 2000 May 13;355(9216):1684–7. 10.1016/S0140-6736(00)02240-6 10905244

[37] Alabi O, Doctor HV, Jumare A, Sahabi N, Abdulwahab A, Findley SE, et al. Health & demographic surveillance system profile: the Nahuche Health and Demographic Surveillance System, northern Nigeria (Nahuche HDSS). Int J Epidemiol. 2014 Dec;43(6):1770–80. 10.1093/ije/dyu197 25399021

[38] Natukwatsa D, Wosu AC, Ndyomugyenyi DB, Waibi M, Kajungu D. An assessment of noncommunicable disease mortality among adults in Eastern Uganda, 2010–2016. PLOS ONE. 2021 Mar 19;16(3):e0248966. 10.1371/journal.pone.0248966 33739993 PMC7978282

[39] Newberry Le Vay J, Fraser A, Byass P, Tollman S, Kahn K, D’Ambruoso L, et al. Mortality trends and access to care for cardiovascular diseases in Agincourt, rural South Africa: a mixed-methods analysis of verbal autopsy data. BMJ Open. 2021 Jun 25;11(6):e048592. 10.1136/bmjopen-2020-048592 34172550 PMC8237742

[40] Fenta EH, Sisay BG, Gebreyesus SH, Endris BS. Trends and causes of adult mortality from 2007 to 2017 using verbal autopsy method, Addis Ababa, Ethiopia. BMJ Open. 2021 Nov 16;11(11):e047095. 10.1136/bmjopen-2020-047095 34785542 PMC8596056

[41] Joshi R, Cardona M, Iyengar S, Sukumar A, Raju CR, Raju KR, et al. Chronic diseases now a leading cause of death in rural India – mortality data from the Andhra Pradesh Rural Health Initiative. Int J Epidemiol. 2006 Dec;35(6):1522–9. 10.1093/ije/dyl168 16997852

[42] Alam N, Chowdhury HR, Ahmed A, Rahman M, Streatfield PK. Distribution of cause of death in rural Bangladesh during 2003–2010: evidence from two rural areas within Matlab Health and Demographic Surveillance site. Glob Health Action. 2014 a Oct 29;7(1):25510. 10.3402/gha.v7.25510 25377333 PMC4220145

[43] Madhavan SR, Reddy S, Panuganti PK, Joshi R, Mallidi J, Raju K, et al. Epidemiology of sudden cardiac death in rural South India – insights from the Andhra Pradesh Rural Health Initiative. Indian Pacing Electrophysiol J. 2011 Jul;11(4):93–102. 21760680 PMC3128815

[44] Alam N, Chowdhury HR, Das SC, Ashraf A, Streatfield PK. Causes of death in two rural demographic surveillance sites in Bangladesh, 2004–2010: automated coding of verbal autopsies using InterVA-4. Glob Health Action. 2014 b Oct 29;7(1):25511. 10.3402/gha.v7.25511 25377334 PMC4220132

[45] Ke C, Gupta R, Xavier D, Prabhakaran D, Mathur P, Kalkonde YV, et al. Million Death Study Collaborators. Divergent trends in ischaemic heart disease and stroke mortality in India from 2000 to 2015: a nationally representative mortality study. Lancet Glob Health. 2018 Aug;6(8):e914–23. 10.1016/S2214-109X(18)30242-0 30012272 PMC6942542

[46] Singh RB, Singh S, Chattopadhya P, Singh K, Singh V, Kulshrestha SK, et al. Tobacco consumption in relation to causes of death in an urban population of north India. Int J Chron Obstruct Pulmon Dis. 2007;2(2):177-85. 18044690 PMC2695616

[47] Saha R, Nath A, Sharma N, Badhan SK, Ingle GK. Changing profile of disease contributing to mortality in a resettlement colony of Delhi. Natl Med J India. 2007 May-Jun;20(3):125–7. 17867616

[48] Wahab A, Choiriyyah I, Wilopo SA. Determining the cause of death: mortality surveillance using verbal autopsy in Indonesia. Am J Trop Med Hyg. 2017 Nov;97(5):1461–8. 10.4269/ajtmh.16-0815 29016331 PMC5817735

[49] Rai SK, Gupta A, Srivastava R, Bairwa M, Misra P, Kant S, et al. Decadal transition of adult mortality pattern at Ballabgarh HDSS: evidence from verbal autopsy data. BMC Public Health. 2015 Aug 14;15(1):781. 10.1186/s12889-015-2119-1 26271623 PMC4536602

[50] Kalkonde Y, Deshmukh M, Kakarmath S, Puthran J, Agavane V, Sahane V, et al. A prospective study of causes of death in rural Gadchiroli, an underdeveloped district of India (2011–2013). J Glob Health Rep. 2019;3:e2019009. 10.29392/joghr.3.e2019009 31909198 PMC6944507

[51] Kanungo S, Tsuzuki A, Deen JL, Lopez AL, Rajendran K, Manna B, et al. Use of verbal autopsy to determine mortality patterns in an urban slum in Kolkata, India. Bull World Health Organ. 2010 Sep 1;88(9):667–74. 10.2471/BLT.09.073742 20865071 PMC2930365

[52] Rai RK, Barik A, Mazumdar S, Chatterjee K, Kalkonde YV, Mathur P, et al. Non-communicable diseases are the leading cause of mortality in rural Birbhum, West Bengal, India: a sex-stratified analysis of verbal autopsies from a prospective cohort, 2012–2017. BMJ Open. 2020 Oct 23;10(10):e036578. 10.1136/bmjopen-2019-036578 33099492 PMC7590361

[53] Shawon MTH, Ashrafi SAA, Azad AK, Firth SM, Chowdhury H, Mswia RG, et al. Routine mortality surveillance to identify the cause of death pattern for out-of-hospital adult (aged 12+ years) deaths in Bangladesh: introduction of automated verbal autopsy. BMC Public Health. 2021 Mar 12;21(1):491. 10.1186/s12889-021-10468-7 33706739 PMC7952220

[54] Phuong Hoa N, Rao C, Hoy DG, Hinh ND, Kim Chuc NT, Ang Ngo D. Mortality measures from sample-based surveillance: evidence of the epidemiological transition in Viet Nam. Bull World Health Organ. 2012 Oct 1;90(10):764–72. 10.2471/BLT.11.100750 23109744 PMC3471050

[55] Huong DL, Minh HV, Vos T, Janlert U, Van DD, Byass P. Burden of premature mortality in rural Vietnam from 1999–2003: analyses from a demographic surveillance site. Popul Health Metr. 2006 Aug 8;4(1):9. 10.1186/1478-7954-4-9 16893472 PMC1559643

[56] Ngo AD, Rao C, Hoa NP, Adair T, Chuc NTK. Mortality patterns in Vietnam, 2006: findings from a national verbal autopsy survey. BMC Res Notes. 2010 Mar 18;3(1):78. 10.1186/1756-0500-3-78 20236551 PMC2851717

[57] Gouda HN, Hazard RH, Maraga S, Flaxman AD, Stewart A, Joseph JC, et al. The epidemiological transition in Papua New Guinea: new evidence from verbal autopsy studies. Int J Epidemiol. 2019 Jun 1;48(3):966–77. 10.1093/ije/dyz018 30915430

[58] Reeve M, Chowdhury H, Mahesh PKB, Jilini G, Jagilly R, Kamoriki B, et al. Generating cause of death information to inform health policy: implementation of an automated verbal autopsy system in the Solomon Islands. BMC Public Health. 2021 Nov 13;21(1):2080. 10.1186/s12889-021-12180-y 34774055 PMC8590305

[59] Abbas SM, Alam AY, Majid A. To determine the probable causes of death in an urban slum community of Pakistan among adults 18 years and above by verbal autopsy. J Pak Med Assoc. 2011 Mar;61(3):235–8. 21465935

[60] Akgün S, Çolak M, Bakar C. Identifying and verifying causes of death in Turkey: national verbal autopsy survey. Public Health. 2012 Feb;126(2):150–8. 10.1016/j.puhe.2011.09.031 22284445

[61] Adair T, Rajasekhar M, Bo KS, Hart J, Kwa V, Mukut MAA, et al. Where there is no hospital: improving the notification of community deaths. BMC Med. 2020 Mar 9;18(1):65. 10.1186/s12916-020-01524-x 32146904 PMC7061465

[62] Leitao J, Desai N, Aleksandrowicz L, Byass P, Miasnikof P, Tollman S, et al. Comparison of physician-certified verbal autopsy with computer-coded verbal autopsy for cause of death assignment in hospitalized patients in low- and middle-income countries: systematic review. BMC Med. 2014 Feb 4;12(1):22. 10.1186/1741-7015-12-22 24495312 PMC3912516

[63] Serina P, Riley I, Hernandez B, Flaxman AD, Praveen D, Tallo V, et al. The paradox of verbal autopsy in cause-of-death assignment: symptom question unreliability but predictive accuracy. Popul Health Metr. 2016 Oct 18;14(1):41. 10.1186/s12963-016-0104-2 27833460 PMC5101673

[64] Thomas L-M, D’Ambruoso L, Balabanova D. Verbal autopsy in health policy and systems: a literature review. BMJ Glob Health. 2018 May 3;3(2):e000639. 10.1136/bmjgh-2017-000639 29736271 PMC5935163

[65] Leitao J, Desai N, Aleksandrowicz L, Byass P, Miasnikof P, Tollman S, et al. Comparison of physician-certified verbal autopsy with computer-coded verbal autopsy for cause of death assignment in hospitalized patients in low- and middle-income countries: systematic review. BMC Med. 2014 Feb 4;12(1):22. 10.1186/1741-7015-12-22 24495312 PMC3912516

[66] Mahesh BPK, Hart JD, Acharya A, Chowdhury HR, Joshi R, Adair T, et al. Validation studies of verbal autopsy methods: a systematic review. BMC Public Health. 2022 Nov 29;22(1):2215. 10.1186/s12889-022-14628-1 36447199 PMC9706899

